# Inbreeding Depression in a Parasitoid Wasp with Single-Locus Complementary Sex Determination

**DOI:** 10.1371/journal.pone.0097733

**Published:** 2014-06-03

**Authors:** Chloé Vayssade, Céline de Fazio, Bastien Quaglietti, Alexandra Auguste, Nicolas Ris, Xavier Fauvergue

**Affiliations:** 1 INRA, UMR 1355 Institute Sophia Agrobiotech, Sophia Antipolis, France; 2 Université de Nice Sophia Antipolis, UMR Institute Sophia Agrobiotech, Sophia Antipolis, France; 3 CNRS, UMR 7254 Institute Sophia Agrobiotech, Sophia Antipolis, France; University of California, Berkeley, United States of America

## Abstract

Inbreeding and inbreeding depression are key processes in small or isolated populations and are therefore central concerns for the management of threatened or (re)introduced organisms. Haplodiploid species of the order Hymenoptera have a particular status with regard to inbreeding depression. Although recessive deleterious alleles that are expressed in males should be purged, an alternative form of inbreeding depression exists in species with single-locus complementary sex determination (sl-CSD). Under sl-CSD, genetically-related parents have a high probability of producing sterile sons instead of fertile daughters. In this article, we study inbreeding depression in *Venturia canescens* (Hymenoptera: Ichneumonidae), a parasitoid wasp with sl-CSD. We used a crossing design to manipulate relatedness according to three levels: within-family, between-family and between-population. For each level, several fitness components were measured on parents and female offspring. We found a 20% reduction in egg load at emergence for inbred crosses. Inbred crosses also yielded a higher proportion of males, as expected in a species with sl-CSD. Mating probability, presence of daughters among offspring, body size, symmetry and longevity were unaffected by inbreeding.

## Introduction

Inbreeding depression is an adverse consequence of inbreeding, *i.e.*, the reproduction of genetically related individuals, which refers to the lower fitness of inbred compared to outbred individuals [1], [2]. Systematic inbreeding occurs routinely in some mating systems, but it should then combine with adaptations alleviating inbreeding depression [3], [4]. In contrast, when mating is random, inbreeding can have dramatic effects. In that case, inbreeding increases with decreasing population size [5], and when it results in inbreeding depression, inbreeding becomes a major component of extinction vortices that threaten small populations [6]–[8]. Inbreeding depression is therefore a process that links population genetics and population dynamics [9], [10] and should, for this reason, be a central concern in population management [11]–[14].

In this paper, we study inbreeding depression in a parasitoid wasp. Planned introductions of parasitoids into novel environments for the biological control of insect pests cause abrupt bottlenecks that impede population establishment [13]. In addition, parasitoids have cycling dynamics resulting from tight demographic feedbacks with their hosts [15] and/or dramatic seasonal variations of environmental factors that yield recurring small population sizes. For these reasons, the study of inbreeding depression in parasitoid wasps is a relevant endeavor from both academic and applied perspectives.

Although superdominance – *i.e.* the higher fitness of heterozygous individuals – and favourable epistasis – *i.e.* a fitness advantage resulting from the interaction among different loci – cannot be excluded [16]–[18], inbreeding depression is mainly due to the expression of recessive deleterious alleles that are brought to a homozygous state by inbreeding [1], [19]. The fixation of such deleterious alleles is commonly detected by comparing the fitness of offspring originating from parents with various degrees of genetic relatedness. Relatedness between parents can be assessed via their pedigree, but access to such information in natural populations is difficult. A widespread alternative is therefore to compare the fitness of offspring from closely related parents (inbred crosses, obtained via selfing or sib mating) to that from parents randomly chosen from either the same population (*e.g.* [20], [21]) or from different populations [9]. Theory predicts that inbreeding should affect life-history traits more than morphological traits [22], and some data are consistent with this prediction (but see [22], [23], [24]).

Parasitoid wasps belong to the Hymenoptera order and are therefore haplodiploid, which raises two interesting twists in the study of inbreeding depression. First, in common with other haplodiploid organisms, parasitoid wasps are expected to be partially immune to inbreeding depression. The reason is that recessive deleterious alleles are exposed to selection at each generation *via* haploid males, so that haplodiploids should suffer a much more benign genetic load than diploids [25]–[27]. Although the purge of deleterious alleles makes sense and is generally supported by data [28]–[31], it does not totally impede inbreeding depression (see for instance [32]). One reason is that the purge should concern neither female-specific genes or traits [33] nor loci with superdominance [34].

The second twist in the study of inbreeding depression in parasitoid wasps comes from the sex determination system of some species of the order Hymenoptera. This system relies on the complementarity of alleles at a single locus and is therefore sensitive to inbreeding. With single-locus complementary sex determination (sl-CSD), individuals that are diploid and heterozygous at the CSD gene develop into females, whereas individuals that are either diploid and homozygote or haploid (and hemizygote) develop into males. Haploid males are normal but diploid males are generally unviable or sterile [35], [36], so that the complementary sex-determiner gene (*csd*) displays an extreme form of superdominance. The production of diploid males being more likely from related parents, sl-CSD is often referred to as a form of inbreeding depression. In small Hymenoptera populations, sl-CSD combined with environmental and demographic stochasticity potentially elevates the base extinction risk in haplodiploids by over an order of magnitude higher than that caused by inbreeding depression in threatened diploids [37]. But what complicates the picture is that sl-CSD favors the evolution of inbreeding avoidance [38]–[40], which in turn increases heterozygosity and may therefore slow down the purging of recessive deleterious alleles involved in female traits.

The solitary parasitoid wasp *Venturia canescens* (Gravenhorst) has a single-locus complementary sex determination system with viable but sterile diploid males [41]. In this species, females partially avoid their brothers for mating [38]. In the field, hosts of *V. canescens* are scarce and dispersed, and parasitism rates are low, so adult parasitoids emerge solitarily; females then search for hosts and males search for females [42], [43]. This suggests a panmictic mating system due to rare encounters between siblings (despite sib-mating avoidance behaviors). Accordingly, genetic analyses with microsatellite markers show no departure from Hardy-Weinberg equilibrium in the studied populations (C. Vayssade, unpublished data). These characteristics, combined with a good knowledge of the species biology [44], [45] make *V. canescens* an appropriate biological model to study inbreeding depression in a species with sl-CSD.

Our objectives are to study inbreeding depression in *V. canescens*, through the production of diploid males and variations in several other fitness traits. To detect inbreeding depression, we performed three types of crosses displaying three levels of relatedness. For each cross, we measured mating probability and offspring sex ratio, as well as several life-history and morphological traits on female offspring, more susceptible to inbreeding depression than haploid males. We expect to observe inbreeding depression shown by a negative relationship between the relatedness of parents and the value of fitness components.

## Materials and Methods

### *Venturia canescens* Strains and Rearing

*Venturia canescens* Gravenhorst (Hymenoptera) is a solitary endoparasitoid of several Lepidoptera species living in dried fruits such as figs, carobs and dates [46]. In the wild, two subspecies of *V. canescens* occur in sympatry. One is asexual and produces only females; the other is sexual and produces both males and females [47]. In the latter, the proportion of males is about 40%. Males can mate several times whereas females are monandrous [38]. In summer 2010, *V. canescens* females from the sexual strain were collected at two locations separated by 230 km and a major mountain range (the Alps). About 150 females were captured in Gotheron, near Valence, France (N 44° 58′ 21″, E 4° 55′ 39″) and 120 females were caught at Mont Boron, near Nice, France (N 43° 41′ 23″, E 7° 18′ 6″). No permission is required to collect samples of this species, which is neither endangered nor protected. These females were used to found two laboratory populations, further referred to as “Valence” and “Nice”. Parasitoids were reared in plastic cages (8×12×25 cm) containing 2^nd^ to 5^th^ instar larvae of the host *Ephestia kuehniella* (Zeller) feeding on organic wheat semolina. Honey and water were applied on the cage net to feed adult wasps. Rearing and experiments were carried out at a temperature of 24±1°C under a LD 16∶8 photoperiod. To limit sib-mating and genetic erosion during rearing (*i.e*., about 4 generations), parasitoids were distributed across several cages and each new generation was initiated with a mix of adults emerging from all the different cages.

### Crossing Method

**Figure 1:**
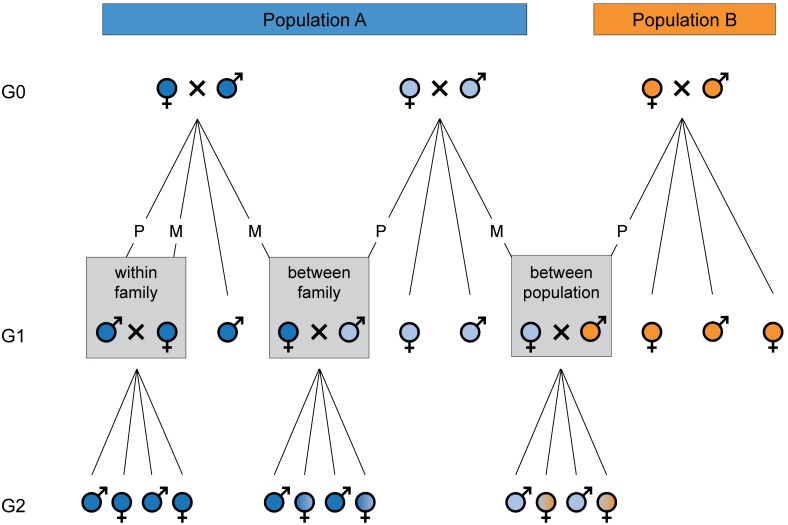
Cross design. At generation G_1_, three types of cross were realized, each based on a different degree of relatedness between the parents. Mating probability and offspring sex ratio were measured on G_1_ individuals. Morphological and life-history traits were measured on individuals of the G_2_ generation. M indicates the maternal family and population, and P the paternal family and population.

To measure the intensity of inbreeding depression on *V. canescens*, we performed crosses with three degrees of relatedness (Fig. 1). At generation G_0_, families were formed by randomly pairing males and females within each population. Families were distributed across four blocks, corresponding to different weeks. Within a block, all pairs were formed the same day. We formed a total of 140 pairs, distributed in 10 pairs per population for blocks 1 to 3, and 40 pairs per population for block 4. At generation G_1_, for each of the 140 families, three virgin females were paired, each with a different male: (1) a brother (within-family cross); (2) an unrelated male from the same population (between-family cross); (3) a male from the other population (between-population cross). Morphological and life-history traits were measured on their female offspring (generation G_2_).

For both G_0_ and G_1_ crosses, parents were collected within 30 minutes following emergence. As mating is scarce during this period, the individuals collected were presumably virgin. For mating and egg-laying, each G_0_ pair was isolated for two days in an individual cage (10×7×2.5 cm) with many hosts. For G_1_ crosses, all females were paired 1–4 days after emergence (*i.e.*, before they become reluctant; [38]). Moreover, the mating protocol for G1 crosses was adapted to allow estimation of mating success: each female was placed in a tube containing hosts and semolina with three males from the same family, a situation promoting mating in *V. canescens*. Individuals were observed 45 minutes or until mating had occurred (*i.e.* when the female remained still and did not reject the male during mounting). As most matings occur within the first ten minutes of observation [38], a 45 min observation time was judged sufficient. The female and the male she mated with (or a randomly selected male if mating had not been observed) were then placed in a vial without hosts in order to increase the probability that females were inseminated. After three days, the female was enclosed for 4 h in a cage containing 100 host larvae at the 3^rd^ or 4^th^ stage to produce the G_2_ generation. Males and females were provided with food and water during all the experiment.

### Variables Measured

To assess the effect of inbreeding on individual fitness, eight variables were measured on either G_1_ parents or G_2_ offspring. On G_1_ (parents), two indices of mating success were assessed: (1) the proportion of females that mated during the 45 min of direct observation and (2) the proportion of females that produced daughters. On G_2_ (offspring), sex ratio, defined as the proportion of males, was measured to detect the production of diploid males, which is expected from brother-sister mating. Moreover, two G_2_ females were randomly selected in each family. One was frozen within 15 min from emergence and served to measure egg load at emergence. The other was kept alive and used to measure egg load at death and longevity. Egg load at emergence estimates the number of eggs a female can lay at the beginning of her adult life. Egg load at death is a measure of the egg storage capacity of a female. To measure egg load, ovaries were dissected under a binocular microscope and mature eggs were counted. Eggs were considered mature if they were in the oviducts, and immature if observed in the ovarioles or calyx. To measure longevity, females were placed in a 10×5 cm tube with water but no food and no host. Survival was checked every two hours between 9∶00 am and 5∶00 pm. The time of death was set as 1∶00 am for females that died overnight. Size and symmetry were measured at emergence and at death. Larger or more symmetrical individuals often have higher fecundity or survival [49], [50]. We measured the length in µm of left (L) and right (R) hind tibias under a binocular microscope (×4) with the software AxioVision version 4.8 (Carl Zeiss). Body size was estimated by the average of L and R, as a correlation between dry body mass and hind tibia length was found in asexual *V. canescens* [48], [51]. The asymmetry *A* of the hind tibias was calculated as A = 2×|L−R|/(L+R).

### Statistical Analyses

Eight response variables were analyzed, either on parents: mating probability, presence of daughters and offspring sex ratio, or on daughters: body size, symmetry, egg load at emergence, egg load at death and longevity. The five explanatory variables were: type of cross, population-arbitrarily defined as the mother’s population for between-population crosses (M; Fig. 1) - maternal family (M; Fig. 1), paternal family (P; Fig. 1) and block. To detect inbreeding depression, the effect of parental relatedness (type of cross) on each response variable was measured by fitting a generalized linear mixed-effects model to the data. Block, maternal family and paternal family were considered as random effects. Maternal and paternal families were nested within block and this structure was accounted for in the models. Type of cross, population, and interaction between cross and population were analyzed as fixed effects. Body size as a main effect or in interaction with cross and population was also included as a fixed effect in analyses of egg load and longevity (in *V. canescens* as in most parasitoids, fecundity and longevity are positively correlated with body size; [45]).

A model with a binomial distribution of errors and a logit link function was used for mating, presence of daughters, and sex ratio. Size and egg load at death were analyzed with a normal distribution and an identity link function, and egg load at emergence with a Poisson distribution and a log link function. Because generalized linear mixed models cannot be implemented with Gamma distributions, log-transformed values of symmetry and longevity were analyzed with a normal distribution and an identity link function. Two data points were excluded from the analyses: a point with extremely high value for egg load at emergence and another with extremely low value for body size in the longevity data set. Including these two outliers would have added, for egg load at emergence, a significant effect of the cross×maternal population interaction (*p* = 0.013) and, for longevity, significant effects of cross (*p* = 0.008) and cross×body size interaction (*p* = 0.006).

For each variable, we first selected the most parsimonious model with regard to random effects. In a second step, we tested the hypotheses of null value of coefficient for each fixed effect. The random effects to be removed from the model were selected by likelihood ratio tests, with the fixed part of the model containing all fixed effects. If all random effects were removed, a standard generalized linear model was fitted. Type III Wald χ^2^ tests were calculated on the model containing the selected random effects and all fixed effects. When tests revealed a significant effect for cross type, least square means (LSM) were estimated for each cross type, in order to document inbreeding depression. Least-squares means were compared pairwise by Z tests and the p-values were adjusted using the Tukey method. All analyses were conducted with the lme4, lmerTest and lsmeans packages in the R statistical software [52]. All values in the text are given as mean ± standard error of the mean (SEM).

## Results

**Figure 2:**
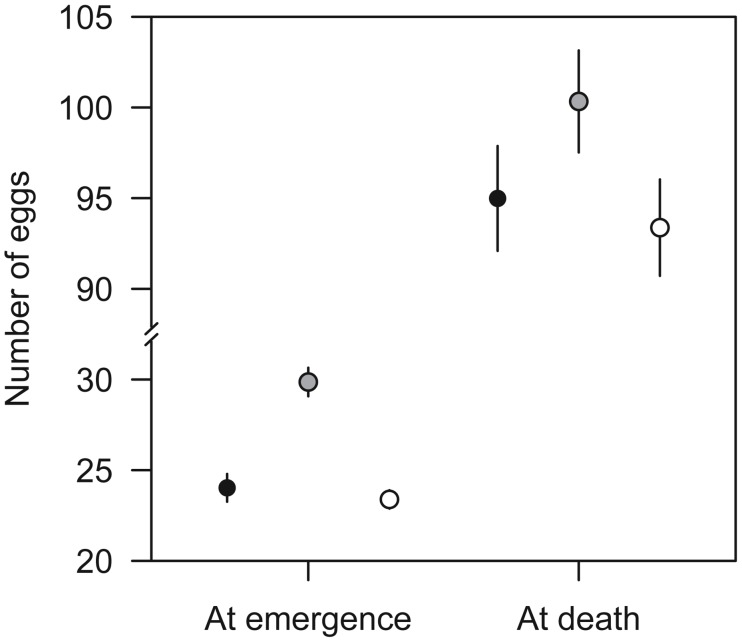
Means ± SEM of egg load at emergence and at death. Means and SEM are calculated for observed values minus the predictions for random effects of a model with cross type, body size and their interaction as fixed effect and paternal and maternal populations as random effects (egg load at emergence) or a model with body size as fixed effect and maternal population and block as random effects (egg load at death). Means for within-family, between-family and between-population crosses are represented by black, grey and empty circles, respectively. For egg load at emergence, least-square means were higher for between-family crosses than for other crosses (within-family/between-family: z = 2.92, *p* = 0.0097; within-family/between-population: z = −0.26, *p* = 0.9627; between-family/between-population: z = 3.14, *p* = 0.0049).

The only strong effect of cross type on offspring fitness was found on egg load at emergence (Table 1A, Fig. 2). Overall, females from between-family crosses had 20% more eggs in their ovarioles (LSM = 29.4) than females from within-family (LSM = 23.8) and between-population (LSM = 23.3) crosses. This effect of cross type was absent for egg load at death (Table 1B), which depended on body size only, although a similar trend was present, with a higher mean egg load for between-family crosses. Egg load at death displayed higher values and within-cross type variance than egg load at emergence (Fig. 2).

**Table 1:** Generalized linear mixed models for (A) egg load at emergence, (B) egg load at death and (C) longevity.

Model	Df	χ^2^	Pr(>χ^2^)
A. Egg load at emergence (Poisson errors, M VC = 0.07, P VC = 0.10, N = 118, n_M_ = 58, n_P_ = 85)			
Cross	2	9.40	0.009
Maternal population	1	0.90	0.342
Body size	1	1.45	0.228
Cross×maternal population	2	5.17	0.076
Cross×body size	2	9.45	0.009
Maternal population×body size	1	0.87	0.352
B. Egg load at death (normal errors, M VC = 191.5, B VC = 252.5, N = 113, n_M_ = 58, n_B_ = 4)			
Cross	2	0.11	0.948
Maternal population	1	0.28	0.598
Body size	1	6.22	0.013
Cross×maternal population	2	0.04	0.980
Cross×body size	2	0.08	0.960
Maternal population×body size	1	0.41	0.523
C. Log(longevity) (normal errors, B VC = 0.05, N = 115, n_B_ = 4)			
Cross	2	4.62	0.099
Maternal population	1	0.32	0.570
Body size	1	3.97	0.046
Cross×maternal population	2	4.33	0.115
Cross×body size	2	4.82	0.090
Maternal population×body size	1	0.29	0.589

For each response variable, details are given in parentheses: error distribution, variance components (VC) for the random effects selected (M = maternal family; P = paternal family; B = block), number of observations (N) and number of levels for random effects (n_M_ = number of maternal families; n_P_ = number of paternal families; n_B_ = number of blocks).

**Figure 3:**
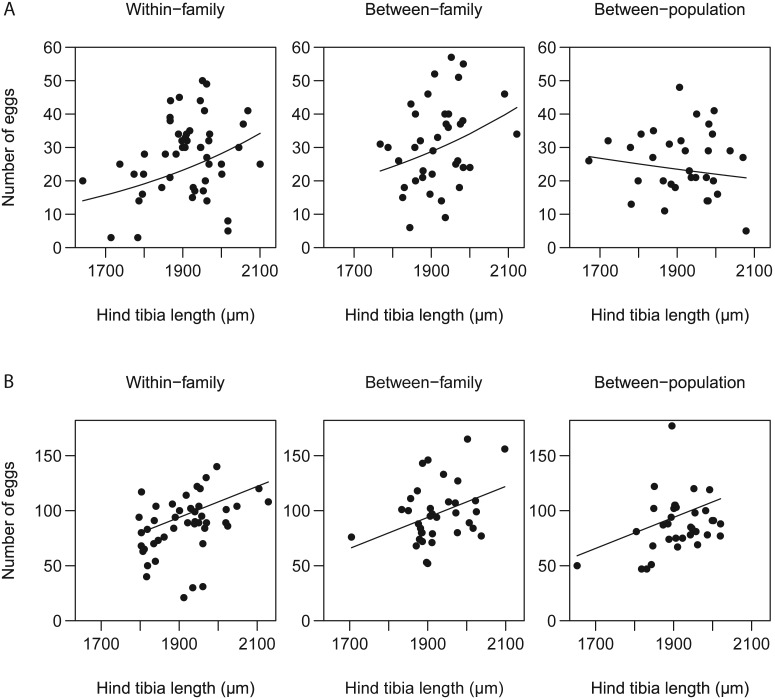
Egg load (A) at emergence and (B) at death as a function of body size for the three cross types. Circles represent observed values and lines represent the predictions of the fixed part of the model with cross type, body size and their interaction as fixed effects and maternal and paternal families as random effects for egg load at emergence, body size as fixed effect and maternal families and block as random effects for egg load at death.

Quite surprisingly, the correlation between egg load at emergence and body size varied according to the type of cross. Egg load at emergence increased with body size for within-family and between-family crosses but not for between-population crosses (Fig. 3A). In contrast, egg load at death increased with body size for all cross types (Fig. 3B).

**Figure 4:**
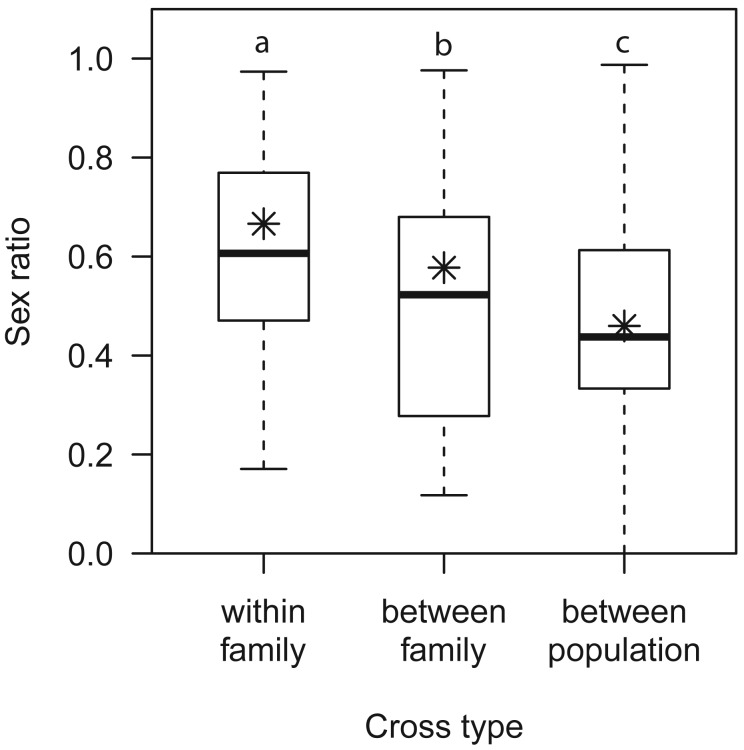
Boxplot of sex ratios for the three cross types. The bottom and top limits of the box are the 0.25 and 0.75 quartiles, respectively, and the bold black line indicates the median. Whiskers represent the minimum and maximum values. Asterisks show represent the predictions of the model with for the type of cross as fixed effect and maternal and paternal families as random effects. Different letters indicate a significant difference of least-square means (within-family/between-family: z = −2.91, *p* = 0.0102; within-family/between-population: z = −6.47, *p*<0.0001; between-family/between-population: z = 4.03, *p* = 0.0002).

The type of cross influenced neither the parent’s probability of mating in 45 min (0.51±0.04, Table 2A), nor the presence of daughters among their progeny (0.68±0.03, Table 2B), but it did influence the offspring sex ratio (Table 2C, Fig. 4). Within-family crosses yielded a higher sex ratio (LSM = 0.67) than between-family (LSM = 0.58) and between-population crosses (LSM = 0.46). Between-family crosses also produced a higher sex ratio than between-population crosses.

**Table 2:** Generalized linear models and generalized linear mixed model for (A) mating probability, (B) presence of daughters among progeny, and (C) offspring sex ratio.

Model	Df	χ^2^	Pr(>χ^2^)
A. Mating probability (binomial errors, N = 176)			
Cross	2	0.29	0.867
Maternal population	1	0.61	0.433
Cross×maternal population	2	3.02	0.221
B. Presence of daughters (binomial errors, N = 251)			
Cross	2	1.85	0.396
Maternal population	1	1.78	0.182
Cross×maternal population	2	2.71	0.258
C. Sex ratio (binomial errors, M VC = 0.74, P VC = 0.86, N = 171, n_M_ = 75, n_P_ = 108)			
Cross	2	10.74	0.005
Maternal population	1	0.37	0.544
Cross×maternal population	2	0.71	0.700

Details are provided in parentheses for each response variable: error distribution, variance components (VC) for the random effects selected (M = maternal family; P = paternal family), number of observations (N) and number of levels for random effects (n_M_ =  number of maternal families; n_P_ = number of paternal families).

The type of cross did not significantly influence body size (1907±7.7 µm, Table 3A) and symmetry of offspring (0.95±0.09, Table 3B), nor did it affect their longevity (109±3.5 h, Table 1C), which was only positively influenced by body size.

**Table 3:** Generalized linear models for (A) body size and (B) symmetry.

Model	Df	F	Pr(>F)
A. Size (normal errors, N = 128)			
Cross	2	1.68	0.192
Maternal population	1	0.91	0.342
Cross×maternal population	2	2.18	0.118
B. log(symmetry) (normal errors, N = 128)			
Cross	2	0.34	0.710
Maternal population	1	0.26	0.611
Cross×maternal population	2	0.40	0.668

For each response variable, details are given in parentheses: error distribution and the number of observations (N).

## Discussion

Some organisms are more sensitive to inbreeding than others [9], [53]. A common assumption is that haplodiploidy alleviates the consequences of inbreeding depression because the expression and subsequent counter-selection of deleterious recessive alleles in haploid males significantly reduces the genetic load. Data tend to support this general expectation: haplodiploid insects and mites do suffer less from inbreeding depression than diploid insects [28]. It is therefore no surprise that archetypal cases of systematic inbreeding are documented in haplodiploids such as pollinating Fig. wasps and parasitoids from the order Hymenoptera [54]–[56].

Careful analyses of inbreeding depression may nevertheless reveal subtle but interesting departures from this widespread view. First, although haplodiploids should be less prone to inbreeding depression, they may not be totally immune to adverse consequences of inbreeding, and both case studies and meta-analyses provide congruent evidence for non-negligible levels of inbreeding depression in haplodiploids [25], [27], [28], [57]. Second, although the purging of load via haploid males may be efficient for genes underpinning many phenotypic traits, it may have no influence on genes that are expressed specifically in females [33]. And beyond haplodiploidy, inbreeding depression is also expected to be higher in life-history traits than in morphological traits [22], [23]. Third, the beneficial effect of the haploid phase of haplodiploids is not expected to impact on alternative mechanisms of inbreeding depression such as superdominance or epistasis. A good example is the single-locus complementary sex determination (sl-CSD) of many hymenopteran insects, which produces a form of inbreeding depression caused by the very low fitness of individuals that are homozygous at the sex determination locus, although no one allele at that locus is in itself deleterious. Such alternative mechanisms may have dramatic consequences in small populations: the extinction risk resulting from sl-CSD in haplodiploids is predicted to be an order of magnitude higher than that produced by inbreeding depression in threatened diploids [37]. Sl-CSD may be the ancestral sex-determination system in Hymenoptera [58], suggesting that other benefits than reduced inbreeding depression (*e.g.* control of sex ratio) have led to the maintenance of haplo-diploidy in Hymenoptera [36]. This sex-determination system adds a fourth twist to the analysis of inbreeding depression in hymenopteran haplodiploids: alternative mechanisms such as sl-CSD promote the evolution of sib mating avoidance [38], [39], which should in turn result in higher heterozygosity at the population level, and potentially, a slower purge of deleterious alleles. Altogether, these different processes tone down the pervasive assumption that inbreeding depression is a minor problem for haplodiploid organisms.

From experimental manipulations of inbreeding coefficients among parents, we reveal here a small but significant level of inbreeding depression in the parasitoid wasp *V. canescens*. Females from sib mating emerged with 20% fewer eggs than females descending from unrelated parents of the same population. In other parasitoid species, inbreeding depression through reduced fecundity was found in two different species of *Trichogramma* [25], [32], *Uscana fumipennis* [28] and *Nasonia vitripennis* [59]. Interestingly, between-population crosses yielded a lower egg load at emergence than between-family crosses, which suggest that both inbreeding depression and outbreeding depression can be at play in *V. canescens*. This pattern fits Bateson’s theory [60] of an optimal genetic distance between parents that maximizes the fitness of offspring. Such reasoning should however be taken with caution given the relatively low genetic and phenotypic distance between the two populations considered. Using 10 microsatellite markers, we indeed found a small but significant Fst of 0.013 between *V. canescens* populations from Nice and Valence (Vayssade, unpublished data).

The egg storage capacity of females, measured by egg load at death, was not affected by inbreeding depression despite inbred females had fewer eggs at emergence. This does not mean that the different genotypes had the same potential fecundity. Indeed, although egg resorption does not occur in *V. canescens* [61], host-deprived females may eject supernumerary eggs, as shown for the asexual subspecies of *V. canescens* [48]. Although both categories of females seem to end up filling their egg storage capacity, inbred females could have a slower egg maturation rate, as suggested by their lower egg load at emergence. Such a situation would be particularly interesting insofar as egg maturation can be traded off with other activities such as flight, which depend on the same energy reserves [62]. Under such a hypothesis, inbred females would have reduced dispersal abilities. Measuring realized fecundity, as was done in other studies showing inbreeding depression for fecundity in parasitoids [25], [28], [32], would provide more information on lifetime number of matured eggs and egg maturation rates.

For offspring of within-family and between-family crosses, we observed a positive correlation between body size and egg load at emergence. Such a relation is well-documented [63]. Here, however, this correlation was absent for between-population crosses. We have no explanation for this and will only mention that Henter [28] found a somewhat similar result with *U. fumipennis:* a positive relationship between body size and fecundity for females from brother-sister crosses but not for females from between-family crosses. Such results underline the necessity to investigate the possible effects of inbreeding depression not only on single traits, but also on correlations among traits.

Contrary to a previous study [38], our measure of mating probability did not highlight a sib-mating avoidance behavior in *V. canescens*, although a tendency to lower mating probability for sib couples was present (mating was observed in 40% of within-family couples *vs.* 58% of within-population couples and 56% of between-population couples). This may be because Metzger *et al.* [38] used only one-day-old females, while we used females aged one to four days. Metzger *et al.* [38] have shown that four-day-old females have a similar mating probability with brothers and unrelated males. It thus seems that the sib-mating avoidance behavior of females disappears between one and four days of age. The female population used in our experiment probably included females both displaying and not displaying this sib-mating avoidance behavior.

Our study adds novel evidence for CSD in *V. canescens* [41]. The sex ratio among offspring increased with increasing genetic relatedness among parents. On average, sibmating yielded an offspring sex ratio of 63%, which is very close to the 65% expected if we assume sl-CSD and 40% unfertilized eggs, as found in *V. canescens* [64]. Although we did not measure the ploidy of male offspring, this change in offspring sex ratio is likely due to a higher proportion of diploid males as a consequence of increased homozygosity. In *V. canescens*, diploid males are similar to haploid males in most respects, but are completely sterile (A. Chuine, C. Vayssade, A. Auguste, E. Desouhant and X. Fauvergue, unpublished data). Hence, the increased proportion of diploid males among offspring with increased genetic relatedness among parents also fits the definition of inbreeding depression.

This study is the first report of inbreeding depression other than the production of diploid males in a parasitoid species with sl-CSD. We detected inbreeding depression through reduced egg load at emergence. Our study also confirmed that sl-CSD is a strong form of inbreeding depression in *V. canescens*. Consequently, in addition to producing less female offspring because of sl-CSD, inbred crosses produce female offspring with a reduced egg load at emergence, although maybe compensated across adult life. *V. canescens* thus seems strongly affected by inbreeding depression.
